# Controlling Growth High Uniformity Indium Selenide (In_2_Se_3_) Nanowires via the Rapid Thermal Annealing Process at Low Temperature

**DOI:** 10.1186/s11671-017-2302-7

**Published:** 2017-09-15

**Authors:** Ya-Chu Hsu, Yu-Chen Hung, Chiu-Yen Wang

**Affiliations:** Department of Material Science and Engineering, National Taiwan University of Science and Technology, Taipei, 10607 Taiwan

**Keywords:** In_2_Se_3_, Nanowire, Rapid thermal annealing (RTA), In situ annealing TEM

## Abstract

High uniformity Au-catalyzed indium selenide (In_2_Se_3)_ nanowires are grown with the rapid thermal annealing (RTA) treatment via the vapor-liquid-solid (VLS) mechanism. The diameters of Au-catalyzed In_2_Se_3_ nanowires could be controlled with varied thicknesses of Au films, and the uniformity of nanowires is improved via a fast pre-annealing rate, 100 °C/s. Comparing with the slower heating rate, 0.1 °C/s, the average diameters and distributions (standard deviation, SD) of In_2_Se_3_ nanowires with and without the RTA process are 97.14 ± 22.95 nm (23.63%) and 119.06 ± 48.75 nm (40.95%), respectively. The in situ annealing TEM is used to study the effect of heating rate on the formation of Au nanoparticles from the as-deposited Au film. The results demonstrate that the average diameters and distributions of Au nanoparticles with and without the RTA process are 19.84 ± 5.96 nm (30.00%) and about 22.06 ± 9.00 nm (40.80%), respectively. It proves that the diameter size, distribution, and uniformity of Au-catalyzed In_2_Se_3_ nanowires are reduced and improved via the RTA pre-treated. The systemic study could help to control the size distribution of other nanomaterials through tuning the annealing rate, temperatures of precursor, and growth substrate to control the size distribution of other nanomaterials.

**Graphical Abstract:**
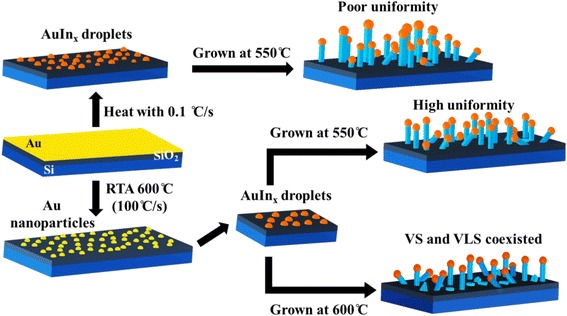
Rapid thermal annealing (RTA) process proved that it can uniform the size distribution of Au nanoparticles, and then it can be used to grow the high uniformity Au-catalyzed In_2_Se_3_ nanowires via the vapor-liquid-solid (VLS) mechanism. Comparing with the general growth condition, the heating rate is slow, 0.1 °C/s, and the growth temperature is a relatively high growth temperature, > 650 °C. RTA pre-treated growth substrate can form smaller and uniform Au nanoparticles to react with the In_2_Se_3_ vapor and produce the high uniformity In_2_Se_3_ nanowires. The in situ annealing TEM is used to realize the effect of heating rate on Au nanoparticle formation from the as-deposited Au film. The byproduct of self-catalyzed In_2_Se_3_ nanoplates can be inhibited by lowering the precursors and growth temperatures.

Rapid thermal annealing (RTA) process proved that it can uniform the size distribution of Au nanoparticles, and then it can be used to grow the high uniformity Au-catalyzed In_2_Se_3_ nanowires via the vapor-liquid-solid (VLS) mechanism. Comparing with the general growth condition, the heating rate is slow, 0.1 °C/s, and the growth temperature is a relatively high growth temperature, > 650 °C. RTA pre-treated growth substrate can form smaller and uniform Au nanoparticles to react with the In_2_Se_3_ vapor and produce the high uniformity In_2_Se_3_ nanowires. The in situ annealing TEM is used to realize the effect of heating rate on Au nanoparticle formation from the as-deposited Au film. The byproduct of self-catalyzed In_2_Se_3_ nanoplates can be inhibited by lowering the precursors and growth temperatures.

## Background

In the past decade, one-dimensional (1D) nanostructure tubes, wires, rods, and belts have become the focal point of the worldwide research in nanotechnology due to their high performance and surface-to-volume ratios, intrinsically associated with low dimensionality, which may lead to unique applications in the various nanoscale device [1, 2]. In particular, 1D semiconductor nanowires (NWs), exhibiting different properties as compared with their bulk or thin film, have shown great potential applications in data storage, computing, and sensing devices [2–4].

Indium selenide (In_2_Se_3_) is a black crystalline and very interesting compound semiconductor of the A^III^B^VI^ group with layered structure, which possessed at least five crystal modifications of α (two-layer hexagonal, 2H), β (three-layer rhombohedral, 3R), γ (defect wurtzite in hexagonal, H), δ, and κ [5, 6]. Due to its polymorphism and the related metal-ion defect structure, In_2_Se_3_ has attracted substantial attention as a promising semiconductor material for several different applications such as photovoltaic solar cell [7, 8], optoelectronics [9], and ionic battery [10].

The layered structure of In_2_Se_3_ is normally consisting of [Se-In-Se-In-Se] sheets stacked with Se atoms along the *c*-axis [11–15]. The strong intralayer bonding and weak interlayer Van der Waals interaction lead to highly anisotropic structural, electrical, optical, and mechanical properties [16, 17]. Layer-structure In_2_Se_3_ nanowires and nanoribbons have been synthesized by using metal nanoparticles as the catalyst via the vapor-liquid-solid (VLS) process [2, 18–20]. The properties of NWs depend not only on their shape anisotropy but also on their crystallographic anisotropy [21]. The vapor-liquid-solid growth mechanism has been demonstrated to control the diameter and growth direction of nanowires [20–24]. Several research results demonstrated that the catalyst is one of an important part to control the morphology of the nanowires. And the crystallographic orientation of a NW is thermodynamically determined at the liquid-solid (LS) interface within the eutectic liquid droplet of a given size and geometry during the initial nucleation [25, 26]. Also, previous studies have shown that the synthesis of highly uniform semiconductor NWs can be achieved through using the well-defined nanoclusters as catalysts in a VLS growth process [25]. Controlling the growth temperature of the Au-In alloy droplet catalyst can determine the segregation concentrations of In and Se atoms in the Au-In alloy droplet which then affects the diameter of the nanowires. However, Au-catalyzed In_2_Se_3_ nanowires are usually grown at relatively high temperature, > 650 °C. According to the Au-In phase diagram, the eutectic temperature is about 530 °C, and the In and/or Se will be precipitated from the Au-In liquid alloy, then reacted with Se to grow the In_2_Se_3_ NWs [27]. In this work, rapid thermal annealing (RTA) is used to make the Au film transfer to uniform Au nanoparticles. Furthermore, lower precursor and growth temperature are chosen to reduce the diameter of nanowires and prevent the VS-grown In_2_Se_3_ byproducts. Interestingly, the thinner In_2_Se_3_ NWs can obtain the diameter by controlling the growth temperature as low as 550 °C. The in situ annealing transmission electron microscopy (TEM) is used to study the effect of heating rate on the Au nanoparticle formation from the as-deposited Au film.

## Experimental

The In_2_Se_3_ NWs were synthesized in a quartz-tube furnace system with a two-temperature zone. Traditionally, the In_2_Se_3_ powder (99.9%, CERAC) was used as a precursor then placed upstream in the middle of the tube at 800 °C (heating rate is 0.01 °C/s). The SiO_2_/Si(100) substrate is coated with a 2.0 nm thick gold film which was placed downstream. The SiO_2_/Si(100) coated with a 2.0-nm-thick gold film is annealed by RTA, at 550 °C (heating rate is 100 °C/s), then the substrate is loaded into the growth furnace tube to grow In_2_Se_3_ nanowires with a flow rate of argon gas at 25 sccm and pressure of 1 Torr. The temperatures of the In_2_Se_3_ precursor powder at the upstream and the Au-coated substrate at the downstream (growth zone) were ramped up to 800 °C (1.2 °C/s) and 550 °C (0.1 °C/s), respectively, and kept for 30 min. The morphologies and microstructure of the In_2_Se_3_ NWs were characterized by scanning electron microscope (SEM, JEOL JSM-6500F) and transmission electron microscopy (TEM, FEI Tecnai™ G^2^ F20 Field Emission Gun) operating at 200 kV. The chemical composition confirmed by energy dispersive X-Ray spectrometer (EDS) is equipped in TEM. The phase of the In_2_Se_3_ NWs is confirmed with an X-ray diffractometer (XRD, D8 DISCOVER SSS Multi-Function High Power). In situ annealing TEM was used to study the effect of heating on Au nanoparticle formation. To prepare in situ heating TEM samples, a 2.0 nm Au film is deposited on a square opening of SiO_2_/Si_3_N_4_ thin film. The thicknesses of the SiO_2_ and Si_3_N_4_ film are 30 and 60 nm, respectively. The 2.0 nm Au film is deposited on the SiO_2_ side, then loaded into TEM to heat with a heating holder (Gatan 652 double tilt heating holder) in the TEM.

## Results and Discussion

**Fig. 1:**
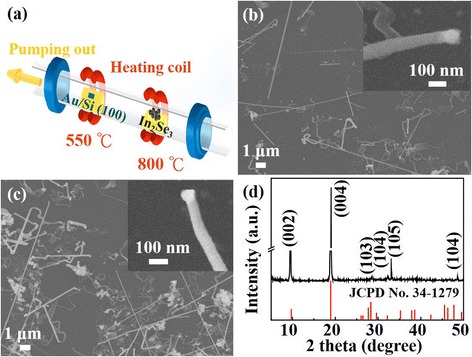
**a** Illustration of a two-zone quartz-tube furnace. The In_2_Se_3_ powder was used as a precursor and placed upstream in the middle of the tube at 800 °C, and SiO_2_/Si(100) coated with a 2.0 nm thick Au film was placed downstream and the argon gas as the carrier gas. **b** and **c** are the SEM images of In_2_Se_3_ nanowires which were grown on the substrate with and without the RTA process, respectively. **d** A typical XRD spectrum of the Au-catalyzed α-In_2_Se_3_ NWs. The lattice constants are *a* = 4.025 Å and *c* = 19.235 Å (JCPDS card, No. 34–1279)

Figure 1a is the schematic illustration of the quartz-tube furnace system that was used to grow the In_2_Se_3_ NWs. Typically, the growth window of Au-catalyzed In_2_Se_3_ NWs is 650–750 °C and the precursor In_2_Se_3_ is heated at 900–950 °C to provide the source of In and Se via a VLS mechanism [19]. However, the Au-In phase diagram shows that the eutectic temperature of Au-In could be as low as 450–550 °C, depending on the composition of the AuIn_x_ alloy [28, 29]. It is expected that the diameters of NWs could be controlled by the Au thickness, growth temperature, and the ambient of the furnace. In this work, the temperatures of the growth temperature and In_2_Se_3_ precursor powder are set as 550 and 800 °C, respectively. Figure 1b, c are the SEM images of the In_2_Se_3_ NWs, grown on the 2.0 nm Au film deposited on a 200-nm SiO_2_/silicon wafer, with and without rapid thermal annealing (RTA) process, respectively. The bright nanoparticle on the top of the NW can be observed from the inset in Fig. 1b, c, which indicates that the In_2_Se_3_ NWs are grown through AuIn_x_ nanoparticles via the VLS mechanism. The average diameters of In_2_Se_3_ NWs (50 nanowires) with and without the RTA process are 97.14 ± 22.95 nm (23.63%) and 119.06 ± 48.75 nm (40.95%), respectively. The average and distribution of the In_2_Se_3_ NW diameters with and without the RTA process are conspicuously different. It clearly exhibits that the RTA process could improve the uniformity and may reduce the diameter of In_2_Se_3_ NWs [30–32]. Figure 1d is the XRD result of In_2_Se_3_ NWs, and all the peaks can be indexed to the hexagonal crystal structure of α-In_2_Se_3_ NWs, in which the lattice constants are *a* = 4.025 Å and *c* = 19.235 Å (JCPDS card, no# 34–1279).

**Fig. 2:**
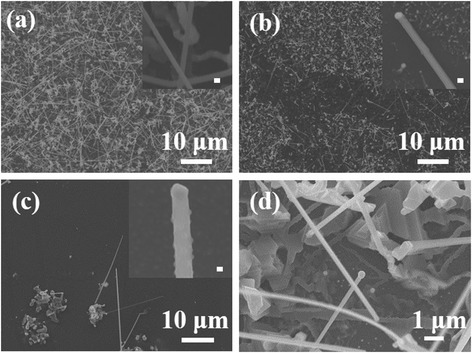
SEM images of In_2_Se_3_ nanowires which were grown at **a** 550 °C, **b** 600 °C, and **c** 650 °C, respectively; the scale bars of the inset images (**a**–**c**) are 100 nm. **d** In_2_Se_3_ nanowires are grown with the precursor and growth temperature at 850 and 600 °C, respectively

Generally, the Au film-coated substrate is loaded into the furnace, the heating rate usually is 1~2 °C/s, then reacts with the precursor to form a low melting point AuIn_x_ alloy, and the In is segregated as the eutectic alloy is supersaturated to react with Se and grow the In_2_Se_3_ NW. The slower heating rate results to poor Au nanoparticle uniformity. Not only the thickness and heating rate of Au film on the substrate but the growth temperature is also an important factor to control the morphology of nanowires. Figure 2a–c are the SEM images of the In_2_Se_3_ NWs after being RTA-treated then grown at 550, 600, and 650 °C, respectively. The corresponding inset images in Fig. 2a–c showed that the In_2_Se_3_ NW diameters were 80–100, 100–200, and 300–500 nm, respectively. The results display that the diameter of In_2_Se_3_ NWs could be tuned by controlling the growth temperature. Since the growth temperature was raised, the In solubility in the Au catalyst would be increased; that means the In atoms need more amounts to reach the supersaturated concentration. In the same time, the thicker In_2_Se_3_ NWs will be grown through the bigger AuIn_x_ droplets. Figure 2d shows the In_2_Se_3_ nanowires grown with the precursor temperature at 850 °C (1.3 °C/s). Both of the Au-catalyzed vapor-liquid-solid growth and self-catalyzed vapor-solid (VS) growth In_2_Se_3_ nanomaterials, including nanowires, nanoplates, and film, will be obtained simultaneously. The higher precursor temperature will lead to the higher precursor vapor, and the excessive precursor will lead to the In_2_Se_3_ product, which tends to self-nucleate and grow. Compared to other studies, the growth temperature, 550 °C, could be much lower than the general reported, 650–750 °C. Furthermore, the precursor temperature could be reduced to 800 °C to prevent self-catalyzed growth. Table 1 lists the comparison of growing In_2_Se_3_ nanowire parameters, including growth substrate temperature (heating rate), growth substrate annealing treatment, precursor temperature, and diameter of nanowires. Due to the lower growth temperature, the byproduct is inhibited such that uniform In_2_Se_3_ NWs could be obtained at a relatively low temperature. It clearly displays that the In_2_Se_3_ NWs could be grown at the lowest growth temperature and precursor temperature in this work. Furthermore, the results of the RTA process showed better diameter uniformity for the In_2_Se_3_ NWs than the conventional system, since the diameter of gold particles were confined.

**Table 1:** Comparison with other works in terms: growth temperature, precursor temperature, and nanowire diameters

Growth temperature (°C)	Diameter (nm)	RTA	Precursor	Precursor temperature (°C)	Reference
650–700	40–80	×	In_2_Se_3_ powder	900–950	[2]
690	80–200	×	In_2_Se_3_ powder	940	[5]
690–740	150	×	In_2_Se_3_ powder	920	[26]
690–740	50–200	×	In_2_Se_3_ powder	920	[33]
690–740	50–200	×	In_2_Se_3_ powder	930–950	[34]
550	70–150	˅	In_2_Se_3_ powder	800	in this work
550	80–170	×	In_2_Se_3_ powder	800	In this work

**Fig. 3:**
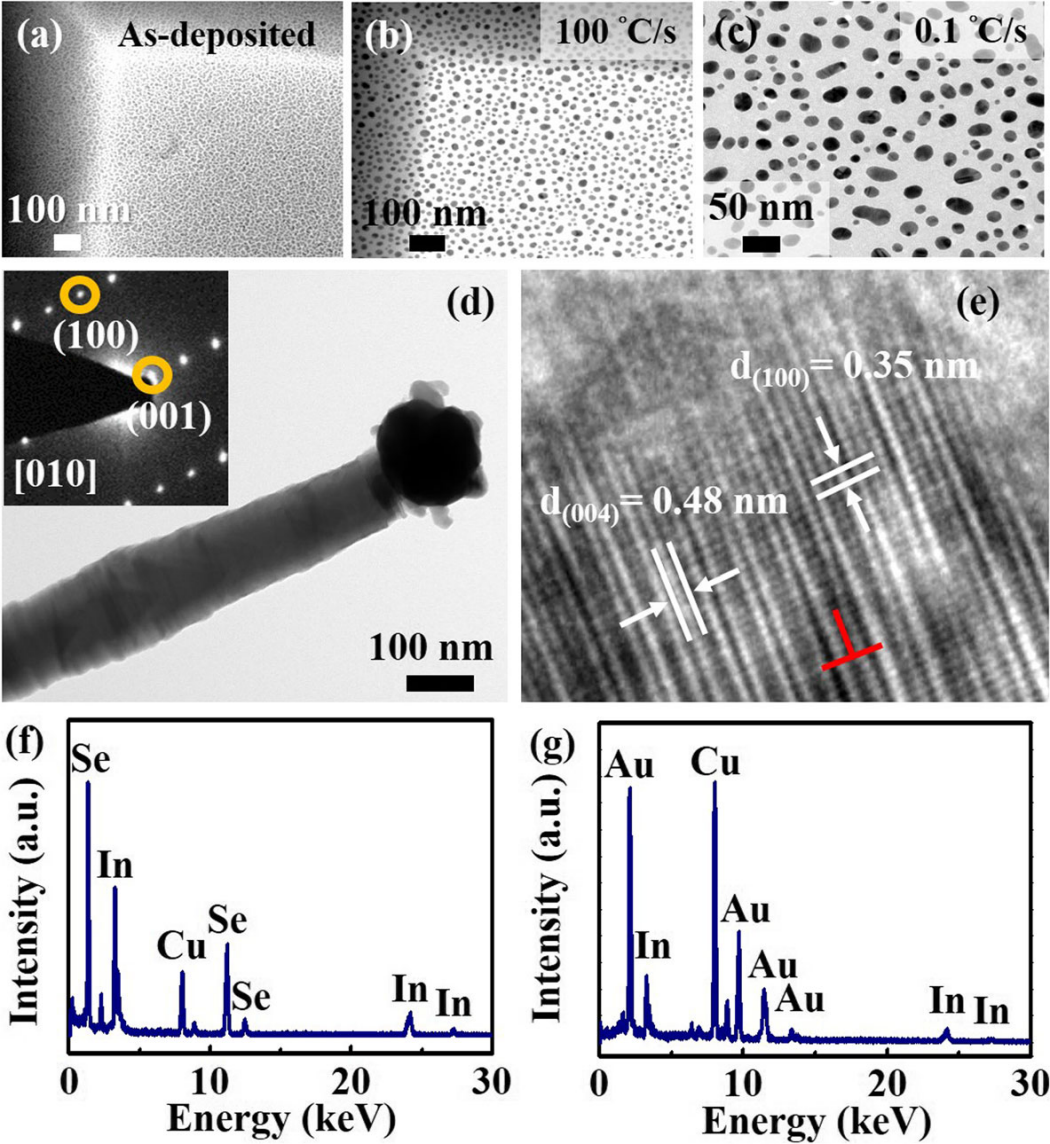
**a** 2.0 nm Au film at room temperature. **b** The gold film by RTA at 550 °C in 100 °C/s. **c** The gold film was ramped up to the 550 °C in 0.1 °C/s. **d** TEM image of an as-synthesized individual α-In_2_Se_3_ nanowire, with an Au nanoparticle tip. SAED pattern of the α-In_2_Se_3_ nanowires (inset). **e** The corresponding HRTEM image of **d** shows the growth direction of the nanowire is along the [001]. **f** and **g** are the EDS spectra of the selected α-In_2_Se_3_ nanowire taken from the body part and the tip part, respectively

In situ annealing TEM is used to study the effect of heating rate on Au nanoparticle formation and nanowire growth. Figure 3a is the TEM image of the as-deposited 2-nm Au film on the SiO_2_/Si_3_N_4_ window, annealing with 0.1 °C/s and 100 °C/s to 550 °C and holding for 30 min. Figure 3b, c are the results of Au nanoparticle formation with the heating rate at 100 °C/s and 0.1 °C/s, respectively. According to the in situ annealing TEM result, Au nanoparticle average size and distribution are analyzed and listed in Table 2. Briefly, the smaller average size and better uniformity of Au nanoparticles could be achieved through the faster heating rate. Figure 3d is the TEM image of a representative In_2_Se_3_ nanowire after being RTA-treated then grown at 550 °C. The result shows that the typical diameter of the nanowire is about 100 nm, and the inset is the corresponding select area electron diffraction (SAED) pattern. Figure 3e shows the high-resolution transmission electron microscopy (HRTEM) image of the corresponding In_2_Se_3_ NW that was taken from the [010] zone axis which has lattice spacing of 0.35 and 0.48 nm and can be indexed to the d-spacing of the (100) and (004) planes, which demonstrates that the In_2_Se_3_ NW is growing along the [001] direction. The EDS analyses are taken from the top and stem; the results are shown in Fig. 3f, g. The Cu and C signals are contributed from the carbon-coated copper TEM grid. Figure 3f which is taken from the stem is composed of In and Se only, and the atomic ratio of In/Se is approximately 2/3. Figure 3g is the EDS result of the top nanoparticle compositions, including In and Au. The additional Au signal proved that the In_2_Se_3_ nanowires are grown via the vapor-liquid-solid (VLS) mechanism. According to the TEM analyses, SEAD, and HRTEM, the VLS-grown nanowires can be identified as α-phase of In_2_Se_3_.

**Table 2:** The average particle size and standard deviation (SD) of Au nanoparticles annealed with RTA (100 °C/s) and without RTA (0.1 °C/s) through the in situ annealing TEM

Annealing condition	Average particle size (nm)	SD (nm)	SD (%)
550_with RTA (100 °C/s)	19.84	5.96	30.00
550_without RTA (0.1 °C/s)	22.06	9.00	40.80

## Conclusions

The lower precursor and growth temperatures 800 and 550 °C, respectively, are provided to grow the Au-catalyzed In_2_Se_3_ nanowires by the VLS mechanism. Furthermore, the uniformity of the In_2_Se_3_ nanowires could be improved by RTA treatment to reduce the size and distribution of Au nanoparticles. The in situ annealing TEM is used to study the effect of heating rate on Au film transfer to Au nanoparticle. The lower precursor and growth temperatures could reduce the formation of self-catalyzed In_2_Se_3_ nanoplates. Lower temperature will lead to lower precursor concentration and low energy, and then the nucleation of self-catalyzed In_2_Se_3_ nanoplates could be inhibited the In_2_Se_3_ nanoplate byproduct.
